# AMPK - a nutrient and energy sensor with roles in diabetes, cancer and viral infection

**DOI:** 10.1186/1753-6561-6-S3-O10

**Published:** 2012-06-01

**Authors:** Grahame Hardie

**Affiliations:** 1 College of Life Sciences, University of Dundee, Dundee, Scotland, UK

## 

The AMP-activated protein kinase (AMPK) is a cellular energy sensor that is conserved throughout the eukaryotic domain. AMPK exists as complexes comprising catalytic α subunits and regulatory β and γ subunits, which are activated >100-fold by phosphorylation of the α subunit at Thr-172 by upstream kinases. Binding of AMP to one site on the Y subunit causes 10-fold allosteric activation by AMP, while binding of either AMP or ADP to a second site promotes phosphorylation and inhibits dephosphorylation of Thr-172. Since both effects are antagonized by ATP, the kinase is activated by falling cellular energy status, when it phosphorylates downstream targets that switch on catabolic pathways generating ATP, while switching off processes consuming ATP. Catabolic processes switched on including uptake and metabolism of glucose and fatty acids, and mitochondrial biogenesis. ATP-consuming processes switched off include biosynthetic pathways for lipids (fatty acids, phospholipids, cholesterol), carbohydrates (gluconeogenesis, glycogen synthesis), proteins and ribosomal RNA, and progress through the cell cycle.

By switching on glucose and fatty acid uptake and metabolism and switching off gluconeogenesis, AMPK should reverse the key metabolic defects in insulin resistance and type 2 diabetes. Consistent with this, AMPK is a target, although probably not the only target, for the anti-diabetic drug metformin. Because AMPK activation inhibits most biosynthetic pathways and causes cell cycle arrest, it should also have a cytostatic, tumor suppressor effect. The discovery in 2003 that the major upstream kinase that phosphorylates Thr-172 is the tumor suppressor kinase, LKB1, strengthened this view. The finding also led to retrospective analyses suggesting that metformin protects against the development of cancer.

If AMPK is a tumor suppressor, one would expect that it would be down-regulated in many tumors. This indeed occurs through loss-of-function mutations in the upstream kinase LKB1, particularly in non-small cell lung cancer and cervical cancer. The majority of cases of breast cancer also appear to have defects in AMPK activation, although the mechanism is not known. We have found that AMPK is down-regulated in cells infected with hepatitis C virus (HCV). HCV may switch off AMPK to prevent its inhibitory effects on lipid and protein synthesis, required for viral replication. HCV infection activates the PKB/Akt pathway, leading to phosphorylation of the α1 subunit of AMPK on Ser-485, thus inhibiting AMPK activation via phosphorylation at Thr-172. It is possible that this contributes to the elevated risk of hepatocellular carcinoma observed in humans with chronic HCV infection.

Finally, we have found that AMPK is directly activated by salicylate, and have evidence that at least some of the metabolic changes induced by the drug in vivo are mediated by AMPK. Salicylate is a natural product of plants whose medicinal use was described in ancient manuscripts. Its use has largely been replaced by derivatives such as salsalate and aspirin, but both are rapidly broken down to salicylate *in vivo.* It is possible that some of the beneficial effects of salicylate derivatives, including amelioration of insulin resistance and protection against colon cancer, might be mediated by AMPK.

